# Molecular characterization of *Spirometra* isolates across the USA

**DOI:** 10.1017/S003118202500054X

**Published:** 2025-04

**Authors:** Tiana L. Sanders, Caroline Sobotyk, Pablo D. Jimenez Castro, Amira Abdu, Jennifer Baade, Mindy Borst, Sriveny Dangoudoubiyam, Brooke A. Delcambre, Jeff M. Gruntmeir, Alice Lee, Christian Leutenegger, Cecilia Lozoya, Gleeson Murphy, Cassan Pulaski, John Schaefer, Adriano Vatta, Heather D. S. Walden, Manigandan Lejeune, Guilherme G. Verocai

**Affiliations:** 1 Department of Veterinary Pathobiology, School of Veterinary Medicine and Biomedical Sciences, Texas A&M University, College Station, TX, USA; 2 Department of Pathobiology, School of Veterinary Medicine, University of Pennsylvania. Philadelphia, PA, USA; 3 Mars Petcare Science & Diagnostics, Antech Diagnostics, Inc., Fountain Valley, CA, USA; 4 Grupo de Parasitología Veterinaria, Universidad Nacional de Colombia, Bogotá, Colombia; 5 Department of Pathobiological Sciences, LSU School of Veterinary Medicine, Louisiana State University, Baton Rouge, LA, USA; 6 Department of Parasitology, Assiut University, Asyut, Egypt; 7 Louisiana Animal Disease Diagnostic Laboratory, Baton Rouge, LA, USA; 8 Lago Vista Animal Clinic, Lago Vista, TX, USA; 9 Texas A&M Veterinary Medical Diagnostic Laboratory, College Station, TX, USA; 10 Department of Comparative Pathobiology, Animal Disease Diagnostic Laboratory, Purdue University College of Veterinary Medicine, West Lafayette, IN, USA; 11 Department of Comparative, Diagnostic and Population Medicine, College of Veterinary Medicine, University of Florida, Gainesville, FL, USA; 12 Diagnostic Bioanalytical and Reagents Laboratory, National Veterinary Services Laboratories, US Department of Agriculture, Ames, IA, USA; 13 Department of Infectious Diseases, College of Veterinary Medicine, University of Georgia, Athens, GA, USA; 14 Department of Biomedical and Diagnostic Services, College of Veterinary Medicine, University of Tennessee, Knoxville, TN, USA; 15 Department of Population Medicine & Diagnostic Sciences, College of Veterinary Medicine Cornell University Ithaca, Ithaca, NY, USA

**Keywords:** Diphyllobothriidea, genetic diversity, North America, sparganosis, species complex, *Spirometra mansoni*, *Spirometra mansonoides*, zoonotic diseases

## Abstract

*Spirometra* is a genus of zoonotic cestodes with an ambiguous species-level taxonomic history. Previously, *Spirometra mansonoides* was considered the only species present in North America. However, recent molecular data revealed the presence of at least three distinct species in the USA: *Spirometra* sp. 2 and 3, and *Spirometra mansoni*. This study aimed to elucidate the diversity and potential host associations of *Spirometra* species among companion animals in the USA. Samples (*N* = 302) were examined from at least 13 host species, including mammals, amphibians and reptiles. Sample types included eggs isolated from faeces (*n* = 222), adult specimens (*n* = 71) and plerocercoids (*n* = 9) from 18 different states and 2 territories across the USA. Extracted genomic DNA was subjected to PCR targeting a fragment of the mitochondrial cytochrome c oxidase subunit 1 (cox1) gene. Generated sequences (*n* = 136) were included in a phylogenetic analysis. *Spirometra mansoni* was detected in domestic cats (*n* = 76), dogs (*n* = 12), a White’s tree frog (*n* = 1), a Cuban knight anole (*n* = 1), a green iguana (*n* = 1) and a serval (*n* = 1) across 15 states and Puerto Rico. *Spirometra* sp. 2 was found only in dogs (*n* = 3) from Florida and *Spirometra* sp. 3 was found only in cats (*n* = 41) from 17 states. All plerocercoid samples were consistent with *S. mansoni*. The results confirm that at least three distinct *Spirometra* species are present and established in companion animals, such as dogs and cats, and likely are using various native and exotic species as paratenic hosts within the USA.

## Graphical abstract

**Figure:**
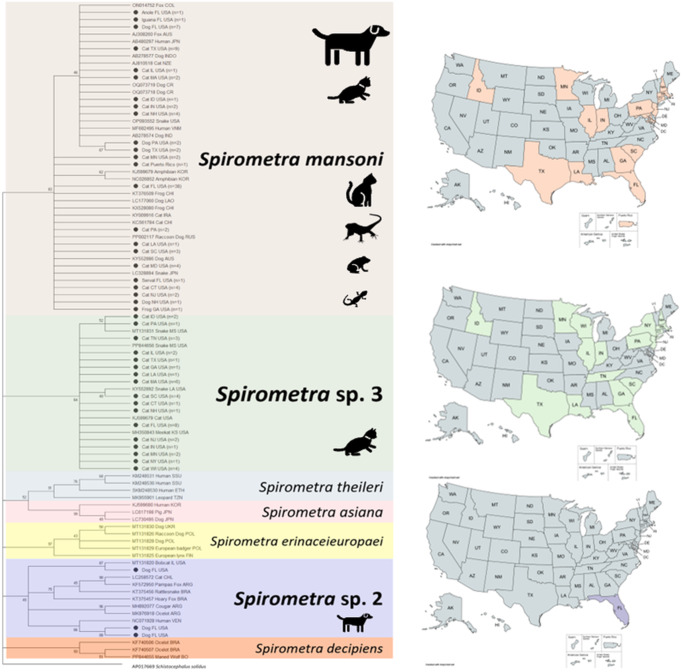

## Introduction

Broad tapeworms within the genus *Spirometra* (Cestoda: Diphyllobothriidea) are known to cause sparganosis (i.e. larval infection), a potentially life-threatening zoonotic disease, and spirometrosis (i.e. adult infection), with reports from every continent except Antarctica (Scholz et al., 2019; Tran et al., 2019; Kuchta et al., 2024). *Spirometra* species are known to parasitize mammals, amphibians, reptiles, birds and rarely fish (Vettorazzi et al., 2023; Kuchta et al., 2024). The lifecycle begins when an infected definitive host defecates faeces containing eggs. The egg must reach freshwater where it hatches and releases a coracidium. The lifecycle requires two intermediate hosts, the first intermediate of which is a freshwater cyclopoid copepod which becomes infected upon the ingestion of a coracidium. The coracidium develops into the first metacestode larval stage, known as the procercoid, within the copepod. The second intermediate host, typically an amphibian or reptilebut occasionally mammals such as humans, become infected upon ingestion of the copepod-containing procercoids; however, experimental studies have shown that infection through direct penetration by the procercoid is also possible (Li, 1929; Mueller, 1938). The procercoid develops into the final metacestode larval stage, the plerocercoid, within the second intermediate host; and this plerocercoid can establish itself in various organs or tissues for several years (Mueller, 1974). Plerocercoids, also termed spargana, can elicit a serious clinical manifestation known as sparganosis within the second intermediate host and a wide range of paratenic hosts. Typically, plerocercoids are located within the subcutaneous tissue, but they can migrate to the viscera, muscles, eyes and central nervous system (Kuchta et al., 2021). The definitive host, typically a carnivoran mammal, becomes infected by ingesting tissues of a paratenic or second intermediate host containing the plerocercoid. The plerocercoid may also infect an individual if exposed to an open wound, for instance, in cases where amphibians such as frogs are used as poultices and placed on the wound (Liu et al., 2015). The plerocercoid develops into an adult cestode within the host’s small intestine and releases eggs into the faeces. In the environment, these eggs embryonate and hatch in freshwater, releasing a coracidium that then completes the life cycle through infection of and development in a freshwater cyclopoid copepod (Mueller, 1974). The prepatent period of this cestode is relatively short with eggs being passed in faeces as early as 2 weeks in cats (Kuchta et al., 2024).

Adult *Spirometra* is generally nonpathogenic within the carnivoran definitive host; however, in some cases, it can cause gastrointestinal disease resulting in vomiting, diarrhoea and weight loss (Conboy, 2009). Infection with the plerocercoid may result in more serious disease and even death depending on the degree of pathology and the tissues involved (Conboy, 2009). In humans, there have been approximately 70 reported cases of sparganosis in North America with the most recent case of a 12-year-old child from Florida in 2024 who presented with a painful subcutaneous mass from which a plerocercoid was surgically excised (Mueller et al., 1963; Taylor, 1976; Griffin et al., 1996; Kuchta et al., 2015; Hawkins et al., 2024). In extreme and rare cases, the plerocercoid asexually reproduces in multiple organs causing a fatal condition known as proliferative sparganosis (Buergelt et al., 1984; Conboy, 2009; Woldemskel, 2014; Tokiwa et al., 2024).

Advancements in molecular diagnostic methods have contributed to the recent reclassification of *Spirometra* species; and, while still controversial, species or lineages are generally confined to specific geographic regions. Kuchta et al. (2024) proposed a new classification scheme based on molecular data and geographic location in which there are seven distinct lineages: *Spirometra erinaceieuropaei* in Europe, *Spirometra theileri* in Africa, *Spirometra asiana* in Korea and Japan, *Spirometra decipiens* in South America, *Spirometra* sp. 2 in both North and South America, *Spirometra* sp. 3 in North America, and *Spirometra mansoni* found worldwide (Yamasaki et al., 2024; Kuchta et al., 2024). Prior to this new classification scheme, *Spirometra* sp. 2 was denoted as *S. decipiens* complex 1 and *Spirometra* sp. 3 was within the *S. decipiens* complex 2. Both species complexes have been molecularly confirmed in North and South America. Kuchta et al. (2024) proposed the *S. decipiens* complex 2 be divided into North and South American lineages with what is now the free-standing *S. decipiens* in South America and *Spirometra* sp. 3 in North America. Additionally, specimens within *S. decipiens* complex 1 are referred to as *Spirometra* sp. 2. This newly proposed naming schematic by Kuchta et al. (2024) is used henceforth.

In North America, the first species to be formally described was named *Diphyllobothrium* (*Spirometra*) *mansonoides*, based on specimens from a domestic cat in the northeastern USA (Mueller, 1935). At the time, *Spirometra* was classified as a subgenus of *Diphyllobothrium*, but it was later elevated to genus (Mueller, 1937; Mueller, 1938). In fact, the first reports of *Spirometra* in the USA pre-date the 1935 description of *S. mansonoides*; in 1927, a cestode was collected from a cat in Louisiana and was morphologically identified as belonging to the genus *Dibothriocephalus*, previously *Diphyllobothrium*, subgenus *Spirometra.* Additionally, tapeworms collected from a cat in Puerto Rico, also in 1927, were classified as *Diphyllobothrium mansoni* (Dikmans, 1931). *Spirometra mansonoides* was originally described based on the morphology of the terminal loops of the uterus. However, this morphological feature is also found within the *S. decipiens* complex and with no supporting molecular data of *S. mansonoides*, it is currently not recognized as a valid species (Kuchta et al., 2024). Nevertheless, *Spirometra* eggs have been routinely observed through faecal examinations of dogs and cats mainly from the eastern USA across decades, and often diagnosed either to genus level or as *S. mansonoides* (Conboy, 2009; Wyrosdick et al., 2017; Hoggard et al., 2019; Nagamori et al., 2020; Sobotyk et al., 2021). Currently, molecular evidence suggests that the *Spirometra* species present in the USA include: *S. mansoni* from a captive Samar cobra (*Naja samarensis*) in Texas, *S. mansoni* from an unknown host in Nebraska, *Spirometra* sp. 2 from a bobcat (*Lynx rufus*) in Illinois, and *Spirometra* sp. 3 from captive meerkats (*Suricata suricatta*) in South Carolina, a black rat snake (*Pantherophis obsoletus*) in Louisiana, an eastern racer snake (*Coluber constrictor*), and a western ribbon snake (*Thamnophis proximus*) both from Mississippi (Jeon et al., 2016; Waeschenbach et al., 2017; McHale et al., 2020; Yamasaki et al., 2021; Verocai et al., 2023; Kuchta et al., 2024).

This study aimed to elucidate the genetic diversity and potential host associations of *Spirometra* cestodes in the USA and its territories through molecular analysis of specimens collected from naturally infected animals and submitted to veterinary diagnostic laboratories nationwide.

## Materials and methods

### Sample acquisition

Samples were requested utilizing the VetPDx (Veterinary Parasitology Diagnostic Network), a listserv founded in 2017 to connect parasitology diagnostic laboratories across North America. A total of 302 samples were received from various locations across the USA, including 25 states and 2 territories (Puerto Rico and the US Virgin Islands). The specimens represented adults (*n* = 71), plerocercoids (*n* = 9), and faecal samples from which *Spirometra* eggs were isolated (*n* = 222). Of the adult specimens, 16 were collected from domestic dogs (*Canis lupus familiaris*), 52 from domestic cats (*Felis silvestris catus*), one from an ocelot (*Leopardus pardalis*), one from a serval (*Leptailurus serval*), and one from an unspecified host. The faecal samples consisted of 29 from domestic dogs, 188 from domestic cats, and five from unspecified hosts. Finally, of the plerocercoids, one was obtained from a green iguana (*Iguana iguana*), one from a rattlesnake of unknown species, one from a wild boar (*Sus scrofa*), one from an opossum (*Didelphis virginiana*), one from a White’s tree frog (*Litoria caerulea*), one from a Cuban knight anole (*Anolis equestris*), and three from New England cottontail rabbits (*Sylvilagus transitionalis*). Overall, samples included both fresh and archival samples from teaching and diagnostic laboratory collections in which samples were collected from 1993 to 2024 in various preservatives such as formalin and ethanol.

### Sample preparation

The different sample types were prepared as follows for DNA extraction: (1) Faecal samples (*n* = 222): The faecal samples were processed by double centrifugation sugar flotation using 2 g of faeces (Hoggard et al., 2019; Zajac et al., 2021). After centrifugation, the top 2 mL of the faecal mixture was siphoned from the 15 mL centrifuge tube and added to a 50 mL conical centrifuge tube. Next, 40 mL of water was added to the tube, vortexed, centrifuged at 400 rcf for 10 min and the supernatant was discarded. This step was repeated. Most of the supernatant was removed, leaving the pellet in approximately 2 mL of water. The pellet and water were then vortexed and strained through a 100 µM ÜberStrainer® (PluriSelect, Leipzig, Germany), followed by straining through a 30 µM ÜberStrainer®. The eggs were then collected from the mesh of the 30 µM ÜberStrainer® and stored in 50 mL conical centrifuge tubes. (2) Adults (*n* = 71) and plerocercoids (*n* = 9): Approximately 1–2 cm of each specimen was placed in a 1.5 mL Eppendorf microtube. The preservative in which each specimen had been kept was then evaporated using a Vacufuge® plus centrifuge concentrator (Eppendorf, Hamburg, Germany) for 6 min.

### Genomic DNA extraction

Genomic DNA was extracted using different methods and kits for the sample types listed. (1) Eggs: For 68 of the 222 samples from which eggs were isolated, an Omni International Bead Ruptor Elite Bead Mill Homogenizer® (Thermo Fisher Scientific, Waltham, MA, USA) was used to break apart the eggs before DNA extraction. To do this, the faecal sediment was added to a 2 mL tube with 1.4 mm ceramic beads and processed with the following settings: 2-min cycle, 10-sec well, 5.00 m sec^−1^, and run for 2 cycles. DNA was then extracted from the samples using the Maxwell® RSC Tissue DNA kit (Promega, Madison, WI, USA) according to the manufacturer’s instructions. The remaining 154 samples containing egg samples were extracted using the Maxwell® RSC Faecal Microbiome DNA kit (Promega, Madison, WI, USA) according to the manufacturer’s instructions. (2) Adult worms and plerocercoids: These were initially beaten with beads in a 2 mL tube with 2.8 mm ceramic beads and processed with the following settings: 2-min cycle, 10-sec dwell, 5.65 m sec^−1^, and run for 2 cycles. DNA was then extracted using the Maxwell® RSC Tissue DNA kit (Promega, Madison, WI, USA) according to the manufacturer’s instructions.

### Molecular analysis

All samples were subjected to polymerase chain reaction (PCR) using primers (forward primer PlatCOI F (5ʹ-TTT TTT GGG CAT CCT GAG GTT TAT-3ʹ) and reverse primer PlatCOI R (5ʹ-TAA AGA AAG AAC ATA ATG AAA ATG-3ʹ)) that targeted a portion of the cytochrome *c* oxidase subunit 1 (*cox1*) gene in the mitochondrial DNA (mtDNA). A modified protocol from Bowles et al. (1995) was utilized to amplify a fragment of the *cox1* gene region in a 25 μL reaction that included Go™ TaqGreen Master Mix (Promega, Madison, WI, USA), 2.5 μL of DNA template and 10 μM of each of primer. Cycling conditions were as follows: denaturation at 95°C for 2 min, followed by 35 cycles of 95°C for 30 sec, 52°C for 1 min, and 72°C for 1 min, followed by a final extension of 72°C for 5 min. DNA extracted from a fragment of an adult *Spirometra* sp. was used as a positive control, and nuclease-free water was used as the negative control. The PCR products were run on a 1.5% agarose gel and visualized using an ultraviolet transilluminator. The expected size of the amplified products was approximately 400 bp in size. Samples that amplified were purified using the E.Z.N.A.® Cycle Pure Kit (Omega Bio–tek, Norcross, GA, USA) and were sequenced in both directions in a 3730xl DNA Analyser at Eurofins Genomics (Louisville, KY, USA).

### Phylogenetic and haplotype analysis

For phylogenetic analysis, sequences were aligned and trimmed using MEGA X version 10.0.5 (Kumar et al., 2018) and were compared to related sequences from NCBI. The phylogenetic tree was constructed from the generated *cox1* gene sequences using the maximum-likelihood method with 1000 bootstrap support and the Tamura-Nei with gamma-distribution (TN93+G) best-fit substitution model (Tamura and Nei, 1993). *Schistocephalus solidus* (AP017669) was used as an outgroup.

For the haplotype network analysis, sequences of *S. mansoni* were analysed. This included 83 sequences (330bp) and 41 reference sequences available in GenBank from Vietnam (*n* = 1), India (*n* = 1), Australia (*n* = 4), Korea (*n* = 2), China (*n* = 3), Iran (*n* = 2), Japan (*n* = 7), Romania (*n* = 1), Indonesia (*n* = 3), New Zealand (*n* = 1), Colombia (*n* = 1), Thailand (*n* = 3), Myanmar (*n* = 2), Tanzania (*n* = 1), Cambodia (*n* = 4), USA (*n* = 1), and Laos (*n* = 4). Nine of the *S. mansoni* sequences generated in our study were removed due to their shorter length. The sequences were prepared in Mega X version 10.0.05 (Kumar et al., 2018) and DnaSP (v6) (Rozas et al., 2017). The prepared sequences were imported into PopART (v1.7) and the median-joining network method was utilized to construct the haplotype network (Bandelt et al., 1999).

## Results

**Figure 1.**
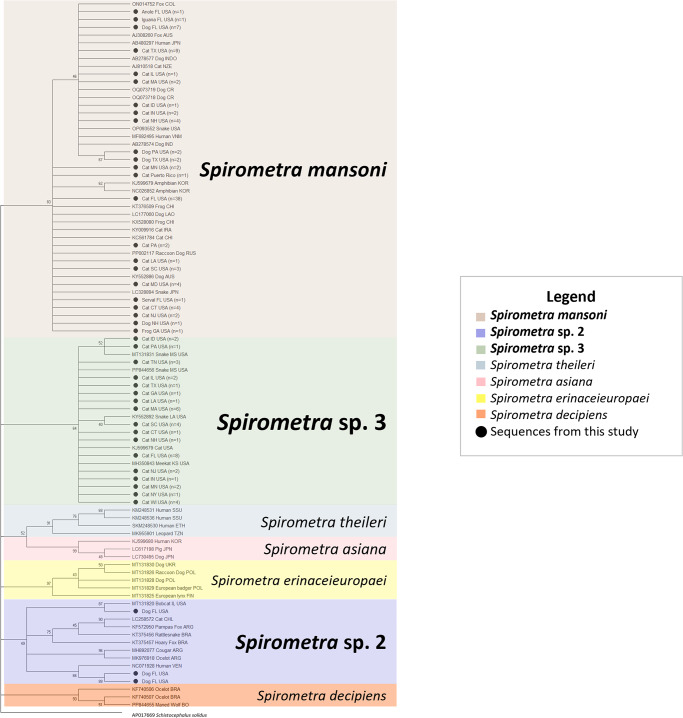
Maximum-likelihood tree inferred from partial *cox1* gene sequences of *Spirometra* samples from this study and related taxa. Sequences from this study are denoted by a solid black circle (●). The best substitution model used was Tamura-Nei + Gamma distribution. *Schistocephalus solidus* was used as outgroup. (AUS – Australia; ARG – Argentina; BO – Bolivia; BRA – Brazil; CHL – Chile; CHI – China; COL – Colombia; CR – Costa Rica; ETH – Ethiopia; FIN – Finland; INDO – Indonesia; IND – India; IRA – Iran; KOR – Korea; JPN – Japan; NZE – New Zealand; PR – Puerto Rico; POL – Poland; RUS – Russia; SSU – South Sudan; TZN – Tanzania; UKR – Ukraine; USA – United States of America; VEN – Venezuela; VNM – Vietnam; CT – Connecticut; FL – Florida; GA – Georgia; IL – Illinois; ID – Idaho; IN – Indiana; KS – Kansas; LA – Louisiana; MA – Massachusetts; MD – Maryland; MN – Minnesota; NJ – New Jersey; NH – New Hampshire; NY – New York; PA – Pennsylvania; SC – South Carolina; TN – Tennessee; TX – Texas; WI – Wisconsin).

**Figure 2.**
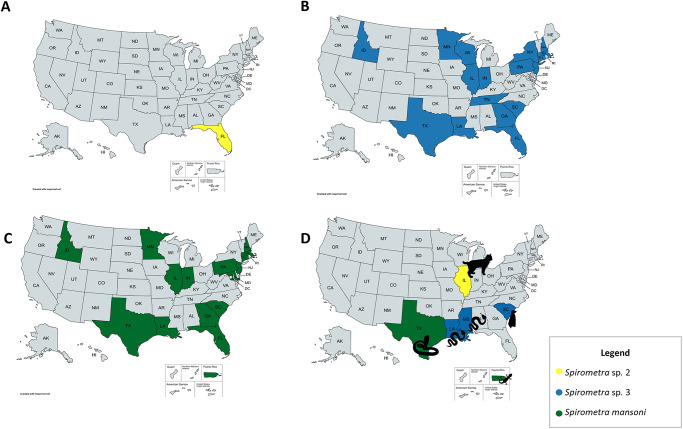
Distribution of *Spirometra* species in the USA. (A) Distribution of *Spirometra* sp. 2. (B) Distribution of *Spirometra* sp. 3. (C) Distribution of *S. mansoni.* (D) Historical distribution of molecularly confirmed cases in the USA with pictograms of host species.

Of the 302 samples received, quality sequences were obtained from 45% (*n* = 136) of them and were included in the phylogenetic analysis (Table 1) and submitted to GenBank (Supplementary Table 1). Of these 136 samples, 83.1% (*n* = 113) came from isolated eggs, 14.7% (*n* = 20) from adult worms, and 2.2% (*n* = 3) from plerocercoids. The phylogenetic analysis indicated three distinct clades among the samples that grouped with previously submitted sequences of *S. mansoni* and *Spirometra* spp. 2 and 3 (Figure 1). Of the 136 sequences, 67.6% (*n* = 92) were most similar to *S. mansoni*, 2.2% (*n* = 3) were similar to *Spirometra* sp. 2, and 30.1% (*n* = 41) were similar to *Spirometra* sp. 3 (Table 1). Within each sample type, the species breakdown is as follows: (1) Of the 113 egg samples, 67.3% (*n* = 76) were identified as *S. mansoni, Spirometra* sp. 3 comprised 32.7% (*n* = 37), and *Spirometra* sp. 2 was not identified in any of the egg samples. (2) Of the 20 adult specimens, 65% (*n* = 13) were identified as *S. mansoni*, 15% (*n* = 3) were identified as *Spirometra* sp. 2, and 20% (*n* = 4) were identified as *Spirometra* sp. 3. (3) Of the plerocercoids, 100% (*n* = 3) were identified as *S. mansoni.* Regarding host association, samples that grouped with *Spirometra* sp. 3 were found only in domestic cats, whereas samples grouping with *Spirometra* sp. 2 were only found in domestic dogs (Table 1). No host association was observed for *S. mansoni.* Geographically, *S. mansoni* was identified in 15 different states and Puerto Rico. Whereas, *Spirometra* sp. 3 was found in 17 different states, and *Spirometra* sp. 2 was restricted to Florida (Figure 2). *Spirometra mansoni* and *Spirometra* sp. 3 had a geographic overlap and were found in 14 of the same states. Additionally, all three lineages were found in Florida.

**Table 1.** Geographic distribution and species identity of samples (*n* = 136) included in the phylogenetic analysis

		*S. mansoni*			*Spirometra* sp. 2	*Spirometra* sp. 3
Origin	Total (n of samples)	Dog	Cat	Other*	Dog	Cat
Connecticut	5	80% (*n* = 4)	NA	NA	NA	20% (*n* = 1)
Florida	59	12% (*n* = 7)	64% (*n* = 38)	5% (*n* = 3)	5% (*n* = 3)	13% (*n* = 8)
Georgia	2	NA	NA	50% (*n* = 1)	NA	50% (*n* = 1)
Idaho	3	NA	33% (*n* = 1)	NA	NA	67% (*n* = 2)
Illinois	3	NA	33% (*n* = 1)	NA	NA	67% (*n* = 2)
Indiana	3	NA	67% (*n* = 2)	NA	NA	33% (*n* = 1)
Louisiana	2	NA	50% (*n* = 1)	NA	NA	50% (*n* = 1)
Massachusetts	8	NA	25% (*n* = 2)	NA	NA	75% (*n* = 6)
Maryland	4	NA	100% (*n* = 4)	NA	NA	NA
Minnesota	4	NA	50% (*n* = 2)	NA	NA	50% (*n* = 2)
New Hampshire	6	17% (*n* = 1)	67% (*n* = 4)	NA	NA	17% (*n* = 1)
New Jersey	4	NA	50% (*n* = 2)	NA	NA	50% (*n* = 2)
New York	1	NA	NA	NA	NA	100% (*n* = 1)
Pennsylvania	5	40% (*n* = 2)	40% (*n* = 2)	NA	NA	20% (*n* = 1)
South Carolina	7	NA	43% (*n* = 3)	NA	NA	57% (*n* = 4)
Tennessee	3	NA	NA	NA	NA	100% (*n* = 3)
Texas	12	17% (*n* = 2)	75% (*n* = 9)	NA	NA	8% (*n* = 1)
Wisconsin	4	NA	NA	NA	NA	100% (*n* = 4)
Puerto Rico	1	NA	100% (*n* = 1)	NA	NA	NA

Percentages are defined as the proportion of samples from a particular state belonging to dog, cat and other* hosts and the *Spirometra* spp. identified.

* Other includes samples from a serval (*Leptailurus serval*), a green iguana (*Iguana iguana*), a White’s tree frog (*Litoria caerulea*) and a Cuban knight anole (*Anolis equestris*).

**Figure 3.**
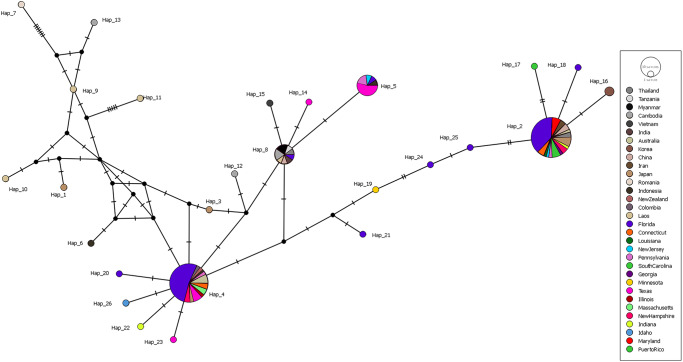
Median-joining haplotype network of *S. mansoni* isolates. A total of 124 sequences, 83 from this study and 41 from GenBank, were included for analysis. The size of the circle corresponds to the number of sequences belonging to each haplotype. The network is color-coded to represent the geographical origins of samples within each haplotype.

Regarding the haplotype network of *S. mansoni*, overall, there were 26 different haplotypes identified from the 124 *cox1* gene sequences (Figure 3). The network analysis indicated two predominant haplotypes to which most of the samples aligned Haplotype 2 (Hap_2) and Haplotype 4 (Hap_4). Hap_2 had a haplotype frequency of 33.9% (*n* = 42) among samples, including samples from Connecticut, Florida, Georgia, Indiana, Louisiana, Maryland, New Hampshire, New Jersey, Pennsylvania, and South Carolina and sequences from Oceania (e.g. Australia) and Asia (e.g. China, Japan, Iran and Thailand). Hap_4 had a frequency of 30.6% (*n* = 38) among samples, including samples from Connecticut, Florida, Illinois, Massachusetts, New Hampshire, Pennsylvania and Texas, and sequences from Oceania (e.g. Australia and New Zealand), Asia (e.g. Japan and Indonesia) and South America (e.g. Colombia). A summary of the geographic origins and haplotype frequencies can be found in Supplementary Table 2.

## Discussion

The genus *Spirometra* is likely the oldest lineage of diphyllobothriidean cestodes; and, since its original description over 200 years ago, the genus has undergone multiple reclassifications (Kuchta et al., 2024). Before this study, the molecular characterization of *Spirometra* isolates from the USA was limited with few studies having been done leading to a significant knowledge gap in species diversity and distribution (Waeschenbach et al., 2017; McHale et al., 2020; Kuchta et al., 2021; Verocai et al., 2023). Despite *S. mansoni* being found worldwide, reports of molecular characterization from cases occurring in the Americas have only been recently described (Brabec et al., 2022; Verocai et al., 2023; Alvarado-Hidalgo et al., 2024; Wu et al., 2024); however, it has not been determined if *S. mansoni* is established and circulating in the Americas, specifically the USA. The first molecular report of *S. mansoni* in the Americas was from a crab-eating fox (*Cerdocyon thous*) in 2022 from Colombia (Brabec et al., 2022; Uribe et al., 2023), followed by a Samar cobra in 2023 from the USA (Verocai et al., 2023). In 2024, there were three published reports that included four dogs, three cats and one coyote (*Canis latrans*) from Costa Rica (Alvarado-Hidalgo et al., 2024), and two Puerto Rican crested anoles (*Anolis cristatellus*) from Puerto Rico (Wu et al., 2024). These cases confirm the presence of *S. mansoni* in the Americas. In the case of the Samar cobra from the USA, which was imported from the Philippines, it was suspected that the snake was infected prior to importation to the USA (Verocai et al., 2023). Additionally, another specimen from an unknown host in Nebraska, USA that was originally labelled as *Spirometra mansonoides* based on morphology was molecularly misidentified as *S. decipiens* and later reclassified as *S. mansoni* (Jeon et al., 2016; Yamasaki et al., 2021, 2024). The results now confirm that *S. mansoni* is likely well established, across the USA, using dogs and cats as definitive hosts, and has had multiple introductions over time, followed by some events of within-country expansion through animal movement. The latter claim is supported by the results of the haplotype network analysis, which indicates intraspecific genetic diversity within *S. mansoni* (Figure 3). While various haplotypes comprised sequences from the USA and other continents, their specific origin cannot be determined.

Regarding *Spirometra* sp. 2, previously named *S. decipiens* complex 1, there has been only a single molecular report from the USA in a bobcat (*L. rufus*), whereas all other reports have originated primarily from both wild and domestic canid and felid hosts in South America (Petrigh et al., 2015; Almeida et al., 2016; Waeschenbach et al., 2017; Arrabal et al., 2020; Fredes et al., 2022; Kuchta et al., 2024). In contrast, samples from this study included only three cases that grouped with *Spirometra* sp. 2, all originating from domestic dogs from Florida (Figure 1). It is plausible that the importation of domestic dogs and cats from South America has led to the establishment of *Spirometra* sp. 2 in North America as well (Jeon et al., 2016). However, *Spirometra* has been reported from a grey fox (*Urocyon cinereoargenteus*) and Florida panthers (*Puma concolor coryi*) (Conti, 1984; Foster et al., 2006), bobcats from Arkansas (Heidt et al., 1988), and coyotes and raccoons (*Procyon lotor*) from various states (Harkema and Miller, 1964; Schaffer et al., 1981; Gompper et al., 2003) without molecular characterization; therefore, it is plausible that *Spirometra* sp. 2 was already established and circulating within the USA. Considering the variety of both wildlife and domestic animal host species that *Spirometra* sp. 2 infects across North and South America, determining any host association within this complex remains challenging. This highlights the need for broader sampling and molecular characterization of specimens from the USA, as well as Central and South America, to better understand the species composition and host associations among different genetic lineages.

Molecular confirmation of *Spirometra* sp. 3, previously within *S. decipiens* complex 2, infections have been reported prior to this study in the USA in reptiles and exotic mammals (Waeschenbach et al., 2017; McHale et al., 2020; Kuchta et al., 2024). In South America, *S. decipiens*, previously within *S. decipiens* complex 2, has been found in a maned wolf (*Chrysocyon brachyurus*) in Bolivia, a Patagonian green racer (*Philodryas patagoniensis*) in Uruguay, and a Geoffroy’s cat (*Leopardus geoffroyi*) and an ocelot (*Leopardus pardalis*) in Brazil (Kuchta et al., 2024). The samples that grouped with *Spirometra* sp. 3 were exclusively detected in domestic cats, suggesting a potential host association within the USA. The sequences from this study represent the first molecular characterization of *Spirometra* isolates from the geographic region in which *S. mansonoides* was first described by Mueller (1935), supporting the idea that *S. mansonoides* belongs in the *Spirometra* sp. 3 Complex. According to Kuchta et al. (2024), isolates within *S. decipiens* complex 2, should be separated into a North and a South American lineage, with those from South America being referred to as *S. decipiens* and those from North America as *Spirometra* sp. 3. The results of this study further support the hypothesis of *S. mansonoides* to be a valid species, possibly represented by *Spirometra* sp. 3 (i.e., Kuchta et al., 2024). Nevertheless, integrated molecular and morphological evidence would be required for resurrecting the species. This could be accomplished by reassessing the type–material of the original *S. mansonoides* description by Mueller (1935), in addition to an attempt of molecular characterization of the material, which may not be fruitful given preservation methods, DNA quality, and destructive use. Alternatively, adult specimens could be collected from the type–host and the type–locality (i.e. Syracuse, New York) to provide a robust molecular characterization followed by phylogenetic analysis for comparison with the data generated by our study.

There are many challenges associated with determining a species based solely on morphology. Historically, the reproductive system of adults was used for identification, specifically the number of uterine coils; however, this can vary as the worm develops and is considered unreliable (Iwata, 1972; Yamasaki et al., 2021). Additionally, the morphology of museum or archival specimens may be distorted, depending on the fixation technique used, and result in misidentification. Furthermore, the opportunistic nature of our sampling limited the ability for morphology to be assessed as the amount of specimen provided was enough only for molecular analysis. Another challenge when working with archival samples, such as the ones obtained in this study, that had an unknown preservation history, is that preservatives such as formalin can greatly inhibit molecular analysis (Zimmermann et al., 2008). Furthermore, most samples included in our study were eggs isolated from faeces, a biological sample known to contain PCR inhibitors such as complex polysaccharides, bilirubin, and metabolites from digestion that may have contributed to the reduced number of high-quality sequences suitable for inclusion in the phylogenetic analysis (Roncancio-Duque et al., 2024). To have a complete taxonomic description, future studies should focus on both molecular and morphologic characterization of larval and adult stages using standardized fixation techniques that are compatible with molecular techniques, (Chávez-González et al., 2022; Chervy, 2024).

This study identified patent infections in both dogs and cats, which may increase the risk of transmission in companion animals and pose an additional threat to public health due to the zoonotic potential of this parasite. Infections with *Spirometra* adults in the small intestine of cats and dogs are typically subclinical, but there are rare instances in which a fatal proliferative sparganosis can occur in companion animals and humans. Proliferative sparganosis develops when the plerocercoids asexually reproduce and migrate into multiple tissues and organs as opposed to non-proliferative infections in which only a few plerocercoids migrate in a confined area within connective tissue (Kikuchi and Maruyama, 2020). Historically, reported cases of symptomatic or fatal proliferative sparganosis in humans or animals, thought to be caused almost exclusively by an isolate phylogenetically closely related to *Spirometra* isolates from South America belonging to *Spirometra* species 2, therefore within the *S. decipiens* complex 1, incorrectly referred to as *Sparganum proliferum* (Miyadera et al., 2001; Kikuchi and Maruyama, 2020; Kikuchi et al., 2021; Fredes et al., 2022); however, a recent case of proliferative sparganosis in a cat in Japan was due to *S. mansoni* (Tokiwa et al., 2024). The Japanese case highlights the importance of determining the taxonomic status and distribution of *Spirometra*, as different species may be associated with a higher degree of pathogenicity. Furthermore, feral cats and other wild carnivoran mammals such as raccoons, may act as reservoirs for *Spirometra* species and potentiate their spread to vulnerable or endangered species in zoological facilities, as was demonstrated by McHale et al. (2020) in which three cases of sparganosis in captive meerkats at a zoological facility was reported.

Although *Spirometra* infections have been reported in Oklahoma, Hawaii, North Carolina and West Virginia, no samples from these areas were available for inclusion in the analysis (Nagamori et al., 2020). Due to the opportunistic and voluntary nature of the sampling strategy employed in the present study, we were unable to confirm the absence of *Spirometra* spp. in states without sample representation. Notwithstanding the limitation of sampling bias, the results still demonstrated an extensive geographic overlap of *S. mansoni* and *Spirometra* sp. 3 with both in 14 of the same states (Figure 2). It is plausible that cats in these states are at an increased risk for co-infection with both lineages. These findings raise the question of how both lineages became established in the same geographic area in the USA. To answer this, we must consider the origins of both the parasite and its host. *Spirometra* isolates within *Spirometra* sp. 3 may have already been present in North America with the bobcat (*L. rufus*), a felid native to North America, serving as the definitive host, and native amphibian and reptile species acting as the second intermediate host (Mueller, 1974). With the colonization of North America by European settlers in the early 16th century, various domestic animals were introduced, including domestic cats. It can be speculated that these animals preyed upon the second intermediate hosts, which led to their infection and integration into the *Spirometra* life cycle (Serpell, 2014).

In contrast, the results indicate that *S. mansoni* is currently well established throughout the USA, and while it is speculated that *S. mansoni* currently has a cosmopolitan distribution with potential origins in Asia, prior to this study there was little molecular evidence to confirm its presence and establishment in North America. Our haplotype analysis further supports the hypothesis of multiple introduction events throughout modern times resulted in the establishment and expansion in the USA. Another explanation, especially pertinent in the last few decades, is the increased travel and rehoming efforts of companion animals such as dogs and cats both domestically and internationally may have further contributed to the introduction of *S. mansoni* into new geographic areas within the USA (Wright et al., 2020; Giannelli et al., 2024). Additionally, the legal and illegal importation of exotic animals to the USA may play an extensive role in modern times in the introduction and spread of zoonotic pathogens such as species of *Spirometra* (Rush et al., 2021, Verocai et al., 2023).

Regarding treatment and prevention, there are currently no FDA-approved products labelled for the treatment of *Spirometra* in companion animals. However, treatment recommendations consist of oral or subcutaneous administration of praziquantel at 25 mg/kg once a day for two consecutive days for both cats and dogs (Conboy, 2009). In cases of proliferative sparganosis, one of the only known successful treatments was of a dog, administered 3 weeks of mebendazole at 20 mg/kg orally once a day followed by 3 weeks of praziquantel at 5 mg/kg orally or subcutaneously once a day, and alternating this regimen for 3 months (Beveridge et al., 1998). Effective prevention strategies must consider the various transmission routes the parasite utilizes to complete the life cycle. A vertebrate host may become infected by (1) ingestion of an infected copepod first intermediate host with a procercoid through contaminated water or food; (ii) ingestion of a second intermediate or paratenic host infected with a plerocercoid; or (iii) migration of a plerocercoid into an open wound of a potential host (Mueller, 1974; Li et al., 2011). Infections in humans primarily occur due to poor food safety and hygiene practices, such as the ingestion of raw or undercooked second intermediate or paratenic hosts, or the use of amphibian poultices on open wounds to facilitate healing in certain cultures (Li et al., 2011; Liu et al., 2015). Education is likely the most vital tool in prevention of human sparganosis and should focus on educating people about how infections occur, emphasizing food safety (i.e. thoroughly cooking meat, especially wildlife), and filtering potentially contaminated drinking water. In regions where the use of frog and snake poultices is common, discouraging this practice and educating about the risk of infection is warranted (Li et al., 2011; Liu et al., 2015). In companion animals, prevention could consist of restricting pets’ access to areas which wildlife inhabit and may contaminate the environment, and monitoring pets to prevent ingestion of potential second intermediate or paratenic hosts. Zoological facilities should consider similar mitigation strategies by implementing surveillance of potential reservoir hosts such as feral cats and raccoons that may contribute to contaminating the environment (McHale et al., 2020).

## Conclusion

*Spirometra mansoni* is well established in the USA and likely has had multiple introductions throughout history. There are two other distinct species of *Spirometra* present within the United States that correspond to *Spirometra* species 2 and 3. Two lineages within *Spirometra* sp. 3 should be considered, one which represents the North American lineage and the other the South American lineage of *Spirometra.* Pending additional integrated classical and molecular assessment, *S. mansonoides* may be resurrected as the taxon shown to infect domestic cats in North America. Overall, this study reinforces the need for further molecular characterization of different *Spirometra* life stages in domestic animals, animals housed in zoological facilities, and wildlife.

## Supporting information

Sanders et al. supplementary material Sanders et al. supplementary material

## Data Availability

Data supporting the conclusions of this study are included in the article. Generated sequences were submitted to GenBank database under accession numbers: PQ673870 – PQ674005.

## Supplementary material

The supplementary material for this article can be found at https://doi.org/10.1017/S003118202500054X.

## References

[ref1] Almeida GG, Coscarelli D, Melo MN, Melo AL and Pinto HA (2016) Molecular identification of *Spirometra* spp. (Cestoda: Diphyllobothriidae) in some wild animals from Brazil. *Parasitology International* 65, 428–431. doi:10.1016/j.parint.2016.05.014 27235572

[ref2] Alvarado-Hidalgo I, Campos-Camacho J, Arguedas-Morales Y, Romero-Vega LM, Alfaro-Alarcón A, Anchia-Ureña G, Bass LG, Berrocal-Ávila I, Hagnauer I, Olivares RWI, Solano-Barquero A, Traube-Rivera R, Montenegro-Hidalgo V and Rojas A (2024) Molecular, morphological, and histopathological evidence of *Spirometra mansoni* in wild and domestic animals from Costa Rica. *Veterinary Parasitology: Regional Studies and Reports* 51. doi:10.1016/j.vprsr.2024.101030 38772646

[ref3] Arrabal JP, Pérez MG, Arce LF and Kamenetsky L (2020) First identification and molecular phylogeny of *Sparganum proliferum* from endangered felid (*Panthera onca*) and other wild definitive hosts in one of the regions with highest worldwide biodiversity. *International Journal of Parasitology: Parasites and Wildlife* 13, 142–149. doi:10.1016/j.ijppaw.2020.09.002 PMC7554206 33083226

[ref4] Bandelt H, Forster P and Röhl A (1999) Median-joining networks for inferring intraspecific phylogenies. *Molecular Biology and Evolution* 16(1), 37–48. 10331250 10.1093/oxfordjournals.molbev.a026036

[ref5] Beveridge I, Friend SE and Jeganathan N (1998) Proliferative sparganosis in Australian dogs. *Australian Veterinary Journal* 76, 757–759. doi:10.1111/j.1751-0813.1998.tb12309.x 9862068

[ref6] Bowles J, Blair D and McManus DP (1995) A molecular phylogeny of the genus. *Echinococcus Parasitology* 110(3), 317–328. doi:10.1017/S0031182000080902 7724239

[ref7] Brabec J, Uribe M, Chaparro-Gutíerrez JJ and Hermosilla C (2022) Presence of *Spirometra mansoni*, causative agent of sparganosis, in South America. *Emerging Infectious Diseases* 28, 2347–2350. doi:10.3201/eid2811.220529 36286232 PMC9622250

[ref8] Buergelt CD, Ellis CG and Senior DF (1984) Proliferative sparganosis in a cat, *The Journal of Parasitology* 70(1), 121. doi:10.2307/3281933 6737156

[ref9] Chávez-González LE, Morales-Calvo F, Mora J, Solano-Barquero A, Verocai GG and Rojas A (2022) What lies behind the curtain: Cryptic diversity in helminth parasites of human and veterinary importance. *Current Research in Parasitology & Vector-Borne Diseases* 2, 000094. doi:10.1016/j.crpvbd.2022.100094 PMC9253710 35800064

[ref10] Chervy L (2024) Manual for the study of tapeworm (Cestoda) parasitic in ray–finned fish, amphibians and reptiles. *Folia Parasitologica* 71, 1–24. doi:10.14411/fp.2024.001 38334295

[ref11] Conboy G (2009) Cestodes of dogs and cats in North America. *Veterinary Clinics: Small Animal Practice* 39(6), 1075–1090. doi:10.1016/j.cvsm.2009.06.005 19932364

[ref12] Conti JA (1984) Helminths of foxes and coyotes in Florida. *Proceedings of the Helminthological Society of Washington* 51, 365–367.

[ref13] Dikmans G (1931) Society Proceedings: Helminthological Society of Washington. *The Journal of Parasitology* 18(1), 44–56.

[ref14] Foster GW, Cunningham MW, Kinsella JM, McLaughlin G and Forrester DJ (2006) Gastrointestinal helminths of free–ranging Florida Panthers (*Puma concolor coryi*) and the efficacy of the current anthelmintic treatment protocol. *Journal of Wildlife Diseases* 42(2), 402–406. doi:10.7589/0090-3558-42.2.402 16870865

[ref15] Fredes F, Mercado R, Salas IP, Sugiyama H, Kobayashi H and Yamasaki H (2022) Morphological observation and molecular phylogeny of *Spirometra decipiens* complex 1 (Cestoda: Diphyllobothriidae) found in cat from Chile. *Parasitology International* 87, 102493. doi:10.1016/j.parint.2021.102493 34737073

[ref16] Giannelli A, Schnyder M, Wright I and Charlier J (2024) Control of companion animal parasites and impact on One Health. *One Health* 18, 100679. doi:10.1016/j.onehlt.2024.100679 39010968 PMC11247265

[ref17] Gompper ME, Goodman RM, Kays RW, Ray JC and Fiorello CV (2003) A survey of the parasites of coyotes (*Canis latrans*) in New York based on faecal analysis. *Journal of Wildlife Diseases* 39(3), 712–717. doi:10.7589/0090-3558-39.3.712 14567236

[ref18] Griffin MP, Tompkins KJ and Ryan MT (1996) Cutaneous Sparganosis. *The American Journal of Dermatopathology* 18(1), 70–72. 8721594 10.1097/00000372-199602000-00011

[ref19] Harkema R and Miller GC (1964) Helminth parasites of the raccoon, *Procyon lotor* in the southeastern United States. *The Journal of Parasitology* 50(1), 60–66. 14125169

[ref20] Hawkins RB, Feely M, Saulino D and Raymond SL (2024) Symptomatic cutaneous sparaganosis (tapeworm) in a child: A case report. *Journal of Pediatric Surgery Case Reports* 110, 102864. doi:10.1016/j.epsc.2024.102864

[ref21] Heidt GA, Rucker RA, Kennedy ML and Baeyena ME (1988) Hematology, intestinal parasites, and selected disease antibodies from a population of bobcats (*Lynx rufus*) in central Arkansas. *Journal of Wildlife Diseases* 24(1), 180–183. doi:10.7589/0090-3558-24.1.180 3352091

[ref22] Hoggard KR, Jarriel DM, Bevelock TJ and Verocai GG (2019) Prevalence survey of gastrointestinal and respiratory parasites of shelter cats in northeastern Georgia, USA. *Veterinary Parasitology Regional Studies and Reports* 16, 100270. doi:10.1016/j.vprsr.2019.100270 31027603

[ref23] Iwata S (1972) Experimental and morphological studies of Manson’s tapeworm, *Diphyllobothrium erinacei*, Rudolphi. Special reference to its scientific name and relationship with *Sparganum proliferum*, Ijima. *Progress Medicine Parasitology Japan* 4, 533–590.

[ref24] Jeon HK, Park H, Lee D, Choe S, Sohn WM and Eom KS (2016) Molecular detection of *Spirometra decipiens* in the United States. *The Korean Journal of Parasitology* 54(4), 503–507. doi:10.3347/kjp.2016.54.4.503 27658603 PMC5040081

[ref25] Kikuchi T, Dayi M, Hunt VL, Ishiwata K, Toyoda A, Kounosu A, Sun S, Maeda Y, Kondo Y, de Noya BA, Noya O, Kojima S, Kuramochi T and Maruyama H (2021) Genome of the fatal tapeworm *Sparganum proliferum* uncovers mechanisms for cryptic life cycle and aberrant larval proliferation. *Communications Biology* 4(1). doi:10.1038/s42003-021-02160-8 PMC8166898 34059788

[ref26] Kikuchi T and Maruyama H (2020) Human proliferative sparganosis update. *Parasitology International* 75, 102036. doi:10.1016/j.parint.2019.102036 31841658

[ref27] Kuchta R, Kołodziej-Sobocńska M, Brabec J, Młocicki D, Sałamatin R and Scholz T (2021) Sparganosis (*Spirometra*) in Europe in the molecular era. *Clinical Infectious Diseases* 72, 882–890. doi:10.1093/cid/ciaa1036 32702118

[ref28] Kuchta R, Phillips AJ and Scholz T (2024) Diversity and biology of *Spirometra* tapeworms (Cestoda: Diphyllobothriidea), zoonotic parasites of wildlife: a review. *International Journal for Parasitology: Parasites and Wildlife* 24. doi:10.1016/j.ijppaw.2024.100947 PMC11261046 39040598

[ref29] Kuchta R, Scholz T, Brabec J and Narduzzi-Wicht B (2015) *Diphyllobothrium, Diplogonoporus* and *Spirometra*. In Xiao L, Ryan U and Feng Y (eds.), *Biology of Foodborne Parasites. Section III Important Foodborne Helminths*. Boca Raton, Florida, USA: CRC Press, pp. 299–326.

[ref30] Kumar S, Stecher G, Li M, Knyaz C and Tamura K (2018) Mega X: Molecular evolutionary genetics analysis across computing platforms. *Molecular Biology and Evolution* 35(6), 1547–1549. doi:10.1093/molbev/msy096 29722887 PMC5967553

[ref31] Li HC (1929) Life histories of *Diphyllobothrium decipiens* and *D. erinacei*. *American Journal of Hygiene* 10, 527–550.

[ref32] Li MW, Song HQ, Li C, Lin HY, Xie WT, Lin RQ and Zhu XQ (2011) Sparganosis in mainland China. *International Journal of Infectious Diseases* 15, 154–156. doi:10.1016/j.ijid.2010.10.001 21126898

[ref33] Liu Q, Li MW, Wang ZD, Zhao GH and Zhu XQ (2015) Human sparganosis, a neglected food borne zoonosis. *The Lancet Infectious Diseases* 15(10), 1226–1235. doi:10.1016/S1473-3099(15)00133-4 26364132

[ref34] McHale B, Callahan RT, Paras KL, Weber M, Kimbrell L, Velázquez–Jiménez Y, McManamon R, Howerth EW and Verocai GG (2020) Sparganosis due to *Spirometra* sp. (Cestoda; Diphyllobothriidae) in captive meerkats (*Suricata suricatta*). *International Journal for Parasitology: Parasites and Wildlife* 13, 186–190. doi:10.1016/j.ijppaw.2023.02.001 33134078 PMC7591330

[ref35] Miyadera H, Kokaze A, Kuramochi T, Kita K, Machinami R, Noya O, Alarcón de Noya B, Okamoto M and Kojima S (2001) Phylogenetic identification of *Sparganum proliferum* as a pseudophyllidean cestode by the sequence analyses on mitochondrial COI and nuclear *sdhB* genes. *Parasitology International* 50, 93–104. doi:10.1016/s1383-5769(01)00071-x 11438431

[ref36] Mueller JF (1935) A Diphyllobothrium from cats and dogs in the Syracuse region. *The Journal of Parasitology* 21, 114–121.

[ref37] Mueller JF (1937) A repartition of the genus *Diphyllobothrium*. *The Journal of Parasitology* 23, 308–310.

[ref38] Mueller JF (1938) The life history of *Diphyllobothrium mansonoides* Mueller, 1935, and some considerations with regard to sparganosis in the United States. *The American Journal of Tropical Medicine* 18, 41–58.

[ref39] Mueller JF (1974) The biology of *Spirometra*. *The Journal of Parasitology* 60(1), 3–14. 4592501

[ref40] Mueller JF, Hart EP and Walsh WP (1963) Human sparganosis in the United States. *The Journal of Parasitology* 49, 294–296.

[ref41] Nagamori Y, Payton ME, Looper E, Apple H, and Johnson EM (2020) Retrospective survey of endoparasitism identified in faeces of client-owned dogs in North America from 2007 through 2018. *Veterinary Parasitology* 282, 109137. doi:10.1016/j.vetpar.2020.109137 32480030

[ref42] Petrigh RS, Scioscia NP, Denegri GM and Fugassa MH (2015) *Cox-1* gene sequences of *Spirometra* in Pampas foxes from Argentina. *Helminthologia* 52(4), 355–359. doi:10.1515/helmin-2015-0056

[ref43] Roncancio-Duque N, García-Ariza JE, Rivera-Franco N, Gonzalez-Ríos AM and López-Alvarez (2024) Comparison of DNA quantity and quality from faecal samples of mammals transported in ethanol or lysis buffer. *One Health* 18, 100731. doi:10.1016/j.onehlt.2024.100731 38655016 PMC11035093

[ref44] Rozas J, Ferrer-Mata A, Sánchez-Del Barrio JC, Guirao-Rico S, Librado P, Ramos-Onsins SE and Sánchez-Gracia A (2017) DnaSP 6: DNA sequence polymorphism analysis of large data sets. *Molecular Biology and Evolution* 34(12), 3299–3302. doi:10.1093/molbev/msx248 29029172

[ref45] Rush ER, Dale E and Aguirre AA (2021) Illegal wildlife trade and emerging infectious diseases: Pervasive impacts to species, ecosystems, and human health. *Animals* 11(6), 1821. doi:10.3390/ani11061821 34207364 PMC8233965

[ref46] Schaffer GD, Davidson WR, Nettles VF and Rollor III EA (1981) Helminth parasites of translocated raccoons (*Procyon lotor*) in the southeastern United States. *Journal of Wildlife Diseases* 17(2), 217–227. doi:10.7589/0090-3558-17.2.217 7241707

[ref47] Scholz T, Kuchta R and Brabec J (2019) Broad tapeworms (Diphyllobothriidae), parasites of wildlife and humans: Recent progress and future challenges. *International Journal for Parasitology: Parasites and Wildlife* 9, 359–369. doi:10.1016/j.ijppaw.2019.02.001 31341771 PMC6630034

[ref48] Serpell JA 2014 Domestication and history of the cat. In Turner DC and Bateson P (eds), *The Domestic Cat: The Biology of Its Behavior*, 3rd edn. Cambridge, UK: Cambridge University Press, pp. 83–99.

[ref49] Sobotyk C, Upton KE, Lejeune M, Nolan TJ, Marsh AE, Herrin BH, Borst MM, Piccione J, Zajac AM, Camp LE, Pulaski CN, Starkey LA, von Simson C and Verocai GG (2021) Retrospective study of canine endoparasites diagnosed by faecal flotation methods analysed across veterinary parasitology diagnostic laboratories, United States, 2018. *Parasites and Vectors* 14, 1–10. doi:10.1186/s13071-021-04960-7 34465379 PMC8406898

[ref50] Tamura K and Nei M (1993) Estimation of the number of nucleotide substitutions in the control region of mitochondrial DNA in humans and chimpanzees. *Molecular Biology and Evolution* 10, 512–526. 8336541 10.1093/oxfordjournals.molbev.a040023

[ref51] Taylor RL (1976) Sparganosis in the United States. *The American Journal of Clinical Pathology* 66, 560–564. 961635 10.1093/ajcp/66.3.560

[ref52] Tokiwa T, Fusimi M, Chou S, Yoshida A, Kinoshita K, Hikima A, Kikuchi T and Ozaki K (2024) Aberrant sparganosis in cat caused by *Spirometra mansoni* (Cestoda: Diphyllothriidae): A case report. *BMC Veterinary Research* 20(148). doi:10.1186/s12917-024-03995-z PMC11031918 38643141

[ref53] Tran QR, Tran MC and Mehanna D (2019) Sparganosis: an under–recognized zoonosis in Australia*?* *BMJ Case Reports.* 12, e228396. doi:10.1136/bcr-2018-228396 PMC6506090 31061178

[ref54] Uribe M, Brabec J, Chaparro–Gutiérrez JJ and Hermosilla C (2023) Neglected zoonotic helminthiases in wild canids: New insights from South America. *Frontiers in Veterinary Science* 10, 1235182. doi:10.3389/fvets.2023.1235182 37635759 PMC10450927

[ref55] Verocai GG, Harvey TV, Sobotyk C, Siu RE, Kulpa M and Connolly M (2023) *Spirometra* infection in a captive Samar cobra (*Naja samarensis*) in the United States: an imported case? *International Journal for Parasitology: Parasites and Wildlife* 20, 133–137. doi:10.1016/j.ijppaw.2023.02.001 36845224 PMC9945636

[ref56] Vettorazzi R, Norbis W, Martorelli SR, García G and Rios N (2023) First report of *Spirometra* (Eucestoda; Diphyllobothriidae) naturally occurring in a fish host. *Folia Parasitologica* 70, 008. doi:10.14411/fp.2023.008 37114794

[ref57] Waeschenbach A, Brabec J, Scholz T, Littlewood DTJ and Kuchta R (2017) The catholic taste of broad tapeworms–multiple routes to human infection. *International Journal for Parasitology* 47(13), 831–843. doi:10.1016/j.ijpara.2017.06.004 28780153

[ref58] Woldemskel M (2014) Subcutaneous sparganosis, a zoonotic cestodiasis, in two cats. *Journal of Veterinary Diagnostic Investigation* 26(2), 316–319. doi:10.1177/1040638713517697 24464556

[ref59] Wright I, Jongejan F, Marcondes M, Peregrine A, Baneth G, Bourdeau P, Bowman DD, Breitschwerdt EB, Capelli G, Cardoso L, Dantas-Torres F, Day MJ, Dobler G, Ferrer L, Gradoni L, Irwin P, Kempf VAJ, Kohn B, Krämer F, Lappin M, Madder M, Maggi RG, Maia C, Miró G, Naucke T, Oliva G, Otranto D, Pennisi MG, Penzhorn BL, Pfeffer M, Roura X, Sainz A, Shin S, Solano-Gallego L, Straubinger RK, Tasker S, Traub R and Little S (2020) Parasites and vector-borne diseases disseminated by rehomed dogs. *Parasites and Vectors* 13, 546. doi:10.1186/s13071-020-04407-5 33168100 PMC7653694

[ref60] Wu TK, Kaneko S, Lucio-Forster A, Spagnoli S, Schultz-Powell L, Liotta J and Bowman D (2024) Cestodiasis in 2 Puerto Rican crested anoles. *Journal of Veterinary Diagnostic Investigation* 36(2), 258–261. doi:10.1177/10406387241229072 38362634 PMC10929623

[ref61] Wyrosdick HM, Chapman A, Martinez J and Schaefer JJ (2017) Parasite prevalence survey in shelter cats in Citrus County, Florida. *Veterinary Parasitology Regional Reports and Studies* 10, 20–24. doi:10.1016/j.vprsr.2017.07.002 31014592

[ref62] Yamasaki H, Sanpool O, Rodpai R, Sadaow L, Laummaunwai P, Un M, Thanchomnang T, Laymanivong S, Aung WPP and Intapan PM (2021) *Spirometra* species from Asia: Genetic diversity and taxonomic challenges. *Parasitology International* 80, 102181. doi:10.1016/j.parint.2020.102181 32898662

[ref63] Yamasaki H, Sugiyama H, Morishima Y and Kobayashi H (2024) Description of *Spirometra asiana* sp. nov. (Cestoda: Diphyllobothriidae) found in wild boars and hound dogs in Japan. *Parasitology International* 98, 102798. doi:10.1016/j.parint.2023.102798 37659580

[ref64] Zajac AM, Conboy GA, Little SE and Reichard MW (2021) *Veterinary Clinical Parasitology*, 9th Edn. New Jersey, USA: John Wiley & Sons, Inc.

[ref65] Zimmermann J, Hajibabaei M, Blackburn DC, Hanken J, Cantin EL, Posfai J and Evans TC Jr (2008) DNA damage in preserved specimens and tissue samples: A molecular assessment. *Frontiers in Zoology* 5, 18. doi:10.1186/1742-9994-5-18 18947416 PMC2579423

