# Supplementary material for: Molecular characterization of Spirometra isolates across the USA
Source: Parasitology. 2025 Apr 16;152(5):477–86. doi: 10.1017/S003118202500054X (PMC12278013; doi:10.1017/S003118202500054X)
Supplement: Sanders et al. supplementary material [file S003118202500054Xsup001.docx]

**Supplementary Table 1**. Description of the 136 samples included in the phylogenetic analysis, including sample host species, geographic location, *Spirometra* species, Sample type, and GenBank accession number.

| Host Species | State | *Spirometra* species | Sample Type | GenBank Accession No. |
| --- | --- | --- | --- | --- |
| *Felis silvestris catus* | CT | *S. mansoni* | Eggs | PQ673870 |
| *Felis silvestris catus* | NJ | *Spirometra* sp. 3 | Eggs | PQ673871 |
| *Felis silvestris catus* | NH | *S. mansoni* | Eggs | PQ673872 |
| *Felis silvestris catus* | CT | *S. mansoni* | Eggs | PQ673873 |
| *Felis silvestris catus* | PA | *S. mansoni* | Eggs | PQ673874 |
| *Felis silvestris catus* | MA | *S. mansoni* | Eggs | PQ673875 |
| *Felis silvestris catus* | NJ | *Spirometra* sp. 3 | Eggs | PQ673876 |
| *Canis lupus familiaris* | PA | *S. mansoni* | Eggs | PQ673877 |
| *Felis silvestris catus* | NY | *Spirometra* sp. 3 | Eggs | PQ673878 |
| *Felis silvestris catus* | CT | *S. mansoni* | Eggs | PQ673879 |
| *Canis lupus familiaris* | PA | *S. mansoni* | Eggs | PQ673880 |
| *Iguana iguana* | FL | *S. mansoni* | Plerocercoid | PQ673881 |
| *Felis silvestris catus* | TN | *Spirometra* sp. 3 | Adult worm | PQ673882 |
| *Felis silvestris catus* | TN | *Spirometra* sp. 3 | Eggs | PQ673883 |
| *Felis silvestris catus* | IN | *Spirometra* sp. 3 | Eggs | PQ673884 |
| *Felis silvestris catus* | NJ | *S. mansoni* | Eggs | PQ673885 |
| *Felis silvestris catus* | TX | *S. mansoni* | Adult worm | PQ673886 |
| *Felis silvestris catus* | PA | *Spirometra* sp. 3 | Eggs | PQ673887 |
| *Felis silvestris catus* | MA | *Spirometra* sp. 3 | Eggs | PQ673888 |
| *Felis silvestris catus* | MA | *Spirometra* sp. 3 | Eggs | PQ673889 |
| *Felis silvestris catus* | TN | *Spirometra* sp. 3 | Eggs | PQ673890 |
| *Felis silvestris catus* | TX | *S. mansoni* | Eggs | PQ673892 |
| *Felis silvestris catus* | TX | *S. mansoni* | Eggs | PQ673893 |
| *Felis silvestris catus* | TX | *S. mansoni* | Eggs | PQ673894 |
| *Felis silvestris catus* | MD | *S. mansoni* | Eggs | PQ673895 |
| *Felis silvestris catus* | SC | *S. mansoni* | Eggs | PQ673896 |
| *Felis silvestris catus* | WI | *Spirometra* sp. 3 | Eggs | PQ673891 |
| *Felis silvestris catus* | LA | *S. mansoni* | Eggs | PQ673898 |
| *Felis silvestris catus* | IN | *S. mansoni* | Eggs | PQ673897 |
| *Felis silvestris catus* | FL | *S. mansoni* | Eggs | PQ673899 |
| *Felis silvestris catus* | FL | *S. mansoni* | Eggs | PQ673900 |
| *Canis lupus familiaris* | FL | *S. mansoni* | Eggs | PQ673901 |
| *Felis silvestris catus* | SC | *Spirometra* sp. 3 | Adult worm | PQ673902 |
| *Felis silvestris catus* | MN | *Spirometra* sp. 3 | Eggs | PQ673903 |
| *Canis lupus familiaris* | FL | *Spirometra* sp. 2 | Adult worm | PQ673904 |
| *Canis lupus familiaris* | FL | *S. mansoni* | Adult worm | PQ673905 |
| *Felis silvestris catus* | PR | *S. mansoni* | Adult worm | PQ673906 |
| *Felis silvestris catus* | MN | *S. mansoni* | Eggs | PQ673907 |
| *Felis silvestris catus* | IL | *Spirometra* sp. 3 | Eggs | PQ673908 |
| *Felis silvestris catus* | FL | *S. mansoni* | Eggs | PQ673909 |
| *Canis lupus familiaris* | FL | *S. mansoni* | Eggs | PQ673910 |
| *Felis silvestris catus* | MA | *S. mansoni* | Eggs | PQ673911 |
| *Felis silvestris catus* | SC | *Spirometra* sp. 3 | Eggs | PQ673912 |
| *Felis silvestris catus* | SC | *S. mansoni* | Eggs | PQ673913 |
| *Felis silvestris catus* | MN | *Spirometra* sp. 3 | Eggs | PQ673914 |
| *Felis silvestris catus* | ID | *S. mansoni* | Eggs | PQ673915 |
| *Felis silvestris catus* | PA | *S. mansoni* | Eggs | PQ673916 |
| *Felis silvestris catus* | SC | *Spirometra* sp. 3 | Eggs | PQ673917 |
| *Felis silvestris catus* | LA | *Spirometra* sp. 3 | Eggs | PQ673918 |
| *Felis silvestris catus* | TX | *Spirometra* sp. 3 | Eggs | PQ673919 |
| *Felis silvestris catus* | NH | *Spirometra* sp. 3 | Eggs | PQ673920 |
| *Felis silvestris catus* | ID | *Spirometra* sp. 3 | Eggs | PQ673921 |
| *Felis silvestris catus* | FL | *S. mansoni* | Eggs | PQ673922 |
| *Felis silvestris catus* | MD | *S. mansoni* | Eggs | PQ673923 |
| *Felis silvestris catus* | TX | *S. mansoni* | Eggs | PQ673924 |
| *Felis silvestris catus* | SC | *S. mansoni* | Eggs | PQ673925 |
| *Canis lupus familiaris* | FL | *S. mansoni* | Eggs | PQ673926 |
| *Felis silvestris catus* | FL | *S. mansoni* | Eggs | PQ673927 |
| *Felis silvestris catus* | WI | *Spirometra* sp. 3 | Eggs | PQ673928 |
| *Felis silvestris catus* | FL | *S. mansoni* | Eggs | PQ673929 |
| *Felis silvestris catus* | FL | *S. mansoni* | Eggs | PQ673930 |
| *Felis silvestris catus* | FL | *S. mansoni* | Eggs | PQ673931 |
| *Felis silvestris catus* | MA | *Spirometra* sp. 3 | Adult worm | PQ673932 |
| *Felis silvestris catus* | IL | *S. mansoni* | Eggs | PQ673933 |
| *Canis lupus familiaris* | TX | *S. mansoni* | Eggs | PQ673934 |
| *Litoria caerulea* | GA | *S. mansoni* | Plerocercoid | PQ673935 |
| *Canis lupus familiaris* | FL | *S. mansoni* | Adult worm | PQ673936 |
| *Felis silvestris catus* | FL | *S. mansoni* | Adult worm | PQ673937 |
| *Anolis equestris* | FL | *S. mansoni* | Plerocercoid | PQ673938 |
| *Felis silvestris catus* | FL | *S. mansoni* | Adult worm | PQ673939 |
| *Leptailurus serval* | FL | *S. mansoni* | Adult worm | PQ673940 |
| *Felis silvestris catus* | FL | *S. mansoni* | Adult worm | PQ673941 |
| *Felis silvestris catus* | FL | *S. mansoni* | Adult worm | PQ673942 |
| *Canis lupus familiaris* | FL | *Spirometra* sp.2 | Adult worm | PQ673943 |
| *Canis lupus familiaris* | FL | *Spirometra* sp. 2 | Adult worm | PQ673944 |
| *Felis silvestris catus* | FL | *S. mansoni* | Adult worm | PQ673945 |
| *Felis silvestris catus* | FL | *S. mansoni* | Adult worm | PQ673946 |
| *Canis lupus familiaris* | TX | *S. mansoni* | Eggs | PQ673947 |
| *Felis silvestris catus* | FL | *S. mansoni* | Eggs | PQ673948 |
| *Felis silvestris catus* | MA | *Spirometra* sp. 3 | Eggs | PQ673949 |
| *Felis silvestris catus* | FL | *S. mansoni* | Eggs | PQ673950 |
| *Felis silvestris catus* | FL | *S. mansoni* | Eggs | PQ673951 |
| *Felis silvestris catus* | WI | *Spirometra* sp. 3 | Eggs | PQ673952 |
| *Felis silvestris catus* | MN | *S. mansoni* | Eggs | PQ673953 |
| *Felis silvestris catus* | FL | *S. mansoni* | Eggs | PQ673954 |
| *Felis silvestris catus* | FL | *S. mansoni* | Eggs | PQ673955 |
| *Felis silvestris catus* | TX | *S. mansoni* | Eggs | PQ673956 |
| *Canis lupus familiaris* | FL | *S. mansoni* | Eggs | PQ673957 |
| *Felis silvestris catus* | FL | *S. mansoni* | Eggs | PQ673958 |
| *Felis silvestris catus* | FL | *S. mansoni* | Eggs | PQ673959 |
| *Felis silvestris catus* | FL | *S. mansoni* | Eggs | PQ673960 |
| *Canis lupus familiaris* | FL | *S. mansoni* | Eggs | PQ673961 |
| *Felis silvestris catus* | SC | *Spirometra* sp. 3 | Eggs | PQ673962 |
| *Felis silvestris catus* | FL | *S. mansoni* | Eggs | PQ673963 |
| *Felis silvestris catus* | FL | *Spirometra* sp. 3 | Eggs | PQ673964 |
| *Felis silvestris catus* | FL | *S. mansoni* | Eggs | PQ673965 |
| *Felis silvestris catus* | MA | *Spirometra* sp. 3 | Eggs | PQ673966 |
| *Felis silvestris catus* | NH | *S. mansoni* | Eggs | PQ673967 |
| *Felis silvestris catus* | TX | *S. mansoni* | Eggs | PQ673968 |
| *Felis silvestris catus* | FL | *S. mansoni* | Eggs | PQ673969 |
| *Felis silvestris catus* | ID | *Spirometra* sp. 3 | Eggs | PQ673970 |
| *Felis silvestris catus* | FL | *S. mansoni* | Eggs | PQ673971 |
| *Felis silvestris catus* | FL | *S. mansoni* | Eggs | PQ673972 |
| *Felis silvestris catus* | CT | *S. mansoni* | Eggs | PQ673973 |
| *Felis silvestris catus* | GA | *Spirometra* sp. 3 | Eggs | PQ673974 |
| *Felis silvestris catus* | FL | *Spirometra* sp. 3 | Eggs | PQ673975 |
| *Felis silvestris catus* | FL | *S. mansoni* | Eggs | PQ673976 |
| *Felis silvestris catus* | NH | *S. mansoni* | Eggs | PQ673977 |
| *Felis silvestris catus* | FL | *Spirometra* sp. 3 | Eggs | PQ673978 |
| *Felis silvestris catus* | FL | *Spirometra* sp. 3 | Eggs | PQ673979 |
| *Felis silvestris catus* | NH | *S. mansoni* | Eggs | PQ673980 |
| *Felis silvestris catus* | WI | *Spirometra* sp. 3 | Eggs | PQ673981 |
| *Felis silvestris catus* | IL | *Spirometra* sp. 3 | Eggs | PQ673982 |
| *Felis silvestris catus* | IN | *S. mansoni* | Eggs | PQ673983 |
| *Felis silvestris catus* | MD | *S. mansoni* | Eggs | PQ673984 |
| *Felis silvestris catus* | FL | *S. mansoni* | Eggs | PQ673985 |
| *Felis silvestris catus* | FL | *S. mansoni* | Eggs | PQ673986 |
| *Felis silvestris catus* | NJ | *S. mansoni* | Eggs | PQ673987 |
| *Felis silvestris catus* | FL | *S. mansoni* | Eggs | PQ673988 |
| *Felis silvestris catus* | FL | *Spirometra* sp. 3 | Eggs | PQ673989 |
| *Felis silvestris catus* | FL | *S. mansoni* | Eggs | PQ673990 |
| *Felis silvestris catus* | FL | *S. mansoni* | Eggs | PQ673991 |
| *Felis silvestris catus* | FL | *Spirometra* sp. 3 | Eggs | PQ673992 |
| *Felis silvestris catus* | TX | *S. mansoni* | Eggs | PQ673993 |
| *Felis silvestris catus* | FL | *S. mansoni* | Eggs | PQ673994 |
| *Felis silvestris catus* | FL | *Spirometra* sp. 3 | Eggs | PQ673995 |
| *Canis lupus familiaris* | NH | *S. mansoni* | Eggs | PQ673996 |
| *Felis silvestris catus* | FL | *S. mansoni* | Eggs | PQ673997 |
| *Felis silvestris catus* | MA | *Spirometra* sp. 3 | Eggs | PQ673998 |
| *Felis silvestris catus* | MD | *S. mansoni* | Eggs | PQ673999 |
| *Felis silvestris catus* | TX | *S. mansoni* | Eggs | PQ674000 |
| *Felis silvestris catus* | CT | *Spirometra* sp. 3 | Eggs | PQ674001 |
| *Felis silvestris catus* | FL | *S. mansoni* | Eggs | PQ674002 |
| *Felis silvestris catus* | FL | *S. mansoni* | Adult worm | PQ674003 |
| *Felis silvestris catus* | FL | *S. mansoni* | Adult worm | PQ674004 |
| *Felis silvestris catus* | FL | *Spirometra* sp. 3 | Adult worm | PQ674005 |

CT: Connecticut, FL: Florida, GA: Georgia, ID: Idaho, IL: Illinois, IN: Indiana, LA: Louisiana, MA: Massachusetts, MD: Maryland, MN: Minnesota, NH: New Hampshire, NJ: New Jersey, NY: New York, PA: Pennsylvania, PR: Puerto Rico, SC: South Carolina, TN: Tennessee, TX: Texas, WI: Wisconsin

Supplementary Table 2: Haplotype frequencies and geographic origins of samples within *Spirometra mansoni*

| Haplotype ID | Haplotype Frequency (%) | Geographic origin of samples |
| --- | --- | --- |
| Hap_1 | 0.8 | JPN |
| Hap_2 | 33.9 | JPN, IR, CHN, AUS, TH, CT, FL, GA, IN, LA, MD, NH, NJ, PA, SC |
| Hap_3 | 0.8 | JPN |
| Hap_4 | 30.6 | JPN, COL, NZ, IDN, AUS, CT, FL, IL, MA, NH, PA, TX |
| Hap_5 | 8.9 | IDN, PA, TX, NJ |
| Hap_6 | 0.8 | IDN |
| Hap_7 | 0.8 | RO |
| Hap_8 | 8.1 | CHN, TH, MMR, IND, TZN, LAO, KHM, FL |
| Hap_9 | 0.8 | LAO |
| Hap_10 | 0.8 | LAO |
| Hap_11 | 0.8 | LAO |
| Hap_12 | 0.8 | KHM |
| Hap_13 | 0.8 | KHM |
| Hap_14 | 0.8 | TX |
| Hap_15 | 0.8 | VN |
| Hap_16 | 1.6 | KOR |
| Hap_17 | 0.8 | PR |
| Hap_18 | 0.8 | FL |
| Hap_19 | 0.8 | MN |
| Hap_20 | 0.8 | FL |
| Hap_21 | 0.8 | FL |
| Hap_22 | 0.8 | IN |
| Hap_23 | 0.8 | TX |
| Hap_24 | 0.8 | FL |
| Hap_25 | 0.8 | FL |
| Hap_26 | 0.8 | ID |

CT: Connecticut, FL: Florida, GA: Georgia, ID: Idaho, IL: Illinois, IN: Indiana, LA: Louisiana, MA: Massachusetts, MD: Maryland, MN: Minnesota, NH: New Hampshire, NJ: New Jersey, PA: Pennsylvania, PR: Puerto Rico, SC: South Carolina, TX: Texas, AUS: Australia, KHM: Cambodia, CHN: China, COL: Colombia, IND: India, IDN: Indonesia, IR: Iran, JPN: Japan, KOR: Korea, LAO: Laos, MMR: Myanmar, NZ: New Zealand, RO: Romania, TZN: Tanzania, TH: Thailand, VN: Vietnam
